# Decreased pH does not alter metamorphosis but compromises juvenile calcification of the tube worm *Hydroides elegans*

**DOI:** 10.1007/s00227-012-2056-9

**Published:** 2012-09-11

**Authors:** Ackley C. Lane, Joy Mukherjee, Vera B. S. Chan, Vengatesen Thiyagarajan

**Affiliations:** The Swire Institute of Marine Science and School of Biological Sciences, The University of Hong Kong, Pok Fu Lam, Hong Kong SAR

## Abstract

Using CO_2_ perturbation experiments, we examined the pre- and post-settlement growth responses of a dominant biofouling tubeworm (*Hydroides elegans*) to a range of pH. In three different experiments, embryos were reared to, or past, metamorphosis in seawater equilibrated to CO_2_ values of about 480 (control), 980, 1,480, and 2,300 μatm resulting in pH values of around 8.1 (control), 7.9, 7.7, and 7.5, respectively. These three decreased pH conditions did not affect either embryo or larval development, but both larval calcification at the time of metamorphosis and early juvenile growth were adversely affected. During the 24-h settlement assay experiment, half of the metamorphosed larvae were unable to calcify tubes at pH 7.9 while almost no tubes were calcified at pH 7.7. Decreased ability to calcify at decreased pH may indicate that these calcifying tubeworms may be one of the highly threatened species in the future ocean.

## Introduction

Carbon dioxide (CO_2_) produced by anthropogenic activity is absorbed by the ocean, reducing the seawater pH and carbonate chemistry that can adversely affect marine organisms, especially calcifiers, many of which are ecological keystones and/or economically important (Widdicombe and Spicer 2008; Doney et al. 2009; Byrne 2011). Given the variable and species-specific responses to low pH, it is important to examine the responses of representative species from as many phyla as possible, especially regarding calcifying organisms (Harley et al. 2006; Ries et al. 2009), for example, the fast growing species that constitute sessile biofouling communities such as barnacles and tubeworms. The majority of sessile marine invertebrates have pelagic larvae specialized for dispersal and the colonization of new habitats. However, planktonic larvae are largely dependent on the pelagic environment to provide the appropriate conditions for growth, that is, any given environmental parameter (e.g., temperature, salinity, pH, and food availability) can turn stressful if conditions are outside larval tolerance ranges (Qiu and Qian 1997). Despite the costs inherent in planktonic life, the necessity of dispersal is more important (Pechenik 1999). After a potentially stressful period of development (from minutes to days), larvae enter a competent stage wherein their primary objective is to attach to a substratum and metamorphose into the juvenile form (Hadfield 2000). The metamorphic process is the pinnacle of the larval phase and as such depends on, and can be affected by, the larval experience up until that point (Thiyagarajan 2010).

Larval attachment, metamorphosis, and subsequent juvenile development of sessile marine invertebrates are dynamic and complicated processes involving the recognition of environmental chemical and physical cues, intensive tissue remodeling, and rapid calcification to form protective shells (Rittschof et al. 1998; Steinberg et al. 2002; Nedved and Hadfield 2009). The vulnerability of larval stages and processes to environmental stressors may extend to pH, possibly being more vulnerable to pH changes than their adult counterparts (Kurihara 2008; Dupont and Thorndyke 2009; Talmage and Gobler 2010; Byrne 2011). The highly complicated process of larval metamorphosis has often been targeted as the weakest link for antifouling compounds and technologies (Rittschof et al. 2011).

Larval life stages differ in more ways than just their degree of exposure to stressors; oftentimes, different life stages have different calcification strategies, for example, the non-calcifying larvae of corals or tube worms, or the primarily chitinous larvae of barnacles that later produce calcareous shell. The larvae of the coral *Porites astreoides*, for example, were unaffected while juvenile growth rates decreased with decreasing pH (Albright et al. 2008). Similarly, the barnacle *Balanus amphitrite* showed only a small decrease in early juvenile shell strength when raised from hatching in decreased pH conditions (McDonald et al. 2009), and while another study observed small decreases in metamorphosis in the same species at the most extreme pH (7.3) (Wong et al. 2011), the effects of decreased pH in barnacles are most obvious during the adult phase, especially when exposed to decreased pH for a longer time, for example, reduced growth, survival, and brood quality of the early juveniles and adults of the temperate (intertidal) barnacle species, *Semibalanus balanoides* (Findlay et al. 2010). The contrast between the larval and juvenile/adult response to decreased pH in corals and (likely) barnacles may be explained by different calcification strategies; coral larvae do not calcify while adults produce aragonite skeletons, and barnacle larvae construct highly chitinous exoskeletons while the juvenile/adult form produces a thick calcitic external shell (Luquet and Marin 2004).In contrast, three copepod species showed high tolerance to decreased pH throughout larval development, into their adult life and even into multiple generations (Mayor et al. 2007; Kurihara and Ishimatsu 2008). Furthermore, molluscan larvae often suffer deleterious effects, having trouble calcifying their aragonitic larval shells, with some species even unable to calcify at all when in decreased pH conditions (e.g., unshelled abalone, Byrne et al. (2011).

Similarly, the biphasic life history of *Hydroides elegans* (Annelida, Polychaeta, Canalipalpata Serpulidae; Haswell, 1883) displays a distinct difference in calcification between larval and adult forms (Nedved and Hadfield 2009). The *H. elegans* is a common biofouling tubeworm used as a model in developmental and biofouling research, which has tolerances to low salinity (~20 ppt), large temperature ranges (15–30 °C), and ranges of food concentrations (developing to settlement with as little as 10^4 ^cells ml^−1^) (Qiu and Qian 1997), The only hard structures produced during larval development are the small, chitinous, setae, whereas the adult produces a large calcareous tube that contains both calcitic and aragonitic forms of calcium carbonate (Tanur et al. 2010; Chan et al. 2012). The aim of this study was to examine the effects of pH on the early life history stages of a tube building polychaete worm, especially interesting considering the contrasting calcification strategies of the pelagic and sessile stages. Expanding on the differences in calcification strategies and associated energetic efforts, the larval phase was expected to be largely unaffected by decreased pH (and decreased carbonate saturation), while the calcifying adult phase was expected to find calcification, and therefore growth, more difficult. Contrary to the evidence supporting this hypothesis, the only other study examining the effects of decreased pH (and copper pollution) on the larvae of a tube-dwelling polychaete (*Pomatoceros lamarckii*) has shown that low pH (7.6 and 7.4) adversely affected larval survival (Lewis et al. 2012). The only other tube-dwelling polychaete observed under low pH conditions (*Spirobis marioni*) was not found at the lower pH stations along a natural pH gradient; however, whether the larvae settled and juveniles could not grow, whether the larvae chose not to settle in low pH areas, or whether the larvae were unable to tolerate, the lower pH is unknown (Cigliano et al. 2010). Here, the *H. elegans* embryos were reared in seawater manipulated by bubbling CO_2_-enriched air to study the larval development, metamorphosis, and juvenile growth in response to pH. Performance was tracked from embryo to 9 days post-metamorphosis in a range of pH environments measuring larval survival, growth, metamorphosis, and juvenile tube length as indicators of physiological condition and overall fitness. Successful attachment and metamorphosis of larvae on suitable substratum and the subsequent rapid juvenile development are important determinants of initiation, establishment, and persistence of marine communities (Qian et al. 2000), and studies such as this contribute greatly to the predictions of future ocean ecologies and biofouling potential of this species.

## Methods

### Study organism, spawning, and larval culture

Adult *Hydroides elegans* were collected from floats on a fish farm in Hong Kong (22°19′N, 114°16′E) on June 1, 2011 (Experiment 1), June 11, 2011 (Experiment 2), and August 9, 2011 (Experiment 3). Adults were conditioned in the laboratory until used (<7 days). Adults were placed in Petri dishes with 0.22-μm filtered seawater (FSW) with 34 ppt salinity and spawning was induced by breaking the tube at room temperature (~24 °C). Gametes from about 25 females and 10 males were collected for each experiment. Fertilization is successful at a broad range of sperm concentrations of greater than 1 × 10^5 ^sperm ml^−1^ (>90 % success), so all eggs were combined with small amounts of sperm from each male (approximate sperm concentration greater than 1 × 10^5 ^sperm ml^−1^) (Pechenik et al. 2007). After 1 h, embryos were concentrated and cleaned using 20-μm mesh and FSW and the total number of eggs was estimated by taking 100 μl aliquots of the homogenous egg mixture. Fertilized eggs were then distributed into the culture tanks described in the experimental designs below. The culture tanks, excepting the differences in pH treatments, were all maintained at 26 °C; salinity was not manipulated but constant at 32–33 ppt; and cultures were fed using concentrated cultures of *Isochryis galbana* resulting in the concentrations of approximately 50,000 cells ml^−1^. This concentration of food is optimal for larval and juvenile growth (Qiu and Qian 1997) and was chosen to maximize larval health, despite the potential for reducing the magnitude of any observed effect.

### Experimental design: pH, ocean acidification, and carbonate system

Seawater partial pressure of carbon dioxide (*p*CO_2_) was raised by bubbling CO_2_-enriched air into the tanks at rates sufficient to achieve the desired pH and carbonate system using the “European Project on Ocean Acidification” (EPOCA) standard procedures (Riebesell et al. 2010). Two to four pH (or *p*CO_2_) treatments, depending on the experiment, were used in this study. Three or four replicate culture tanks were assigned to each pH treatment, covering OA scenarios representing current *p*CO_2_ levels (seawater pH ≈ 8.1) and a range of levels expected to occur over the next 250 years: pH 7.9, pH 7.7, and pH 7.5 (Caldeira and Wickett 2003). The required treatment pH levels were achieved by adjusting the flow rate of air and CO_2_ using dual (air and CO_2_) variable area flow meter/controllers (Cole-Parmer Inc.). The pH was measured twice daily throughout the culture period using a Mettler-Toledo pH meter (NBS scale). Food availability, dissolved oxygen, temperature, and salinity were measured daily. Once in 2 days, total alkalinity was measured using the Apollo SciTech’s AC-A2 Alkalinity Titrator and compared to seawater reference materials (Batch 98, A.G. Dickson, Scripps Institution of Oceanography). The carbonate chemistry equilibrium of each tank was calculated using the CO2SYS program (Pierrot et al. 2006).

### Experiment 1: Effect of pH on embryo and larval development

This experiment was designed to test the effect of pH on embryo and larval development. There were two treatments, pH 8.1 (control) and pH 7.6. Embryos were added into culture tanks with final concentrations of 10 eggs per milliliter. Embryonic developmental success was assessed after 16 h when 50 % of the embryos in the control pH hatched into the trochophore larval stage. After 16 h, undeveloped embryos were removed and hatched larvae were placed in the same treatment cultures containing FSW with algal food (*Isochrysis galbana*). Algal suspensions were maintained at approximate concentrations of 50,000 cells ml^−1^ (Qiu and Qian 1997).

During embryo and larval culture period, the following two measurements were taken. (1) To examine embryo development, the undeveloped embryos and the newly hatched trochophore larvae were filtered out of the tanks after 16 h and concentrated into 100 ml, and from these three, subsamples of 100 μl were taken after mixing the beaker to ensure a homogenous distribution of embryos and larvae. The swimming larvae were then counted in each of the samples and compared to the number of eggs originally added to assess percent embryonic developmental success. (2) Larval size and survivorship measurements were taken when the majority of larvae attained competency to attach and metamorphosis (after ~96 h). Larval size was measured on a compound microscope (Leica DFC 280) with micrometer. Larval survivorship was determined by the percentage of larvae that had achieved competence after 96 h, or the time when approximately 80 % of larvae in the control were competent. Larval competency was judged using their ability to metamorphose on natural biofilms and/or their behavior, that is, at the time of competence larvae generally swim slowly and start crawling close to surface (Qian 1999).

### Experiment 2: Effect of pH on larval metamorphosis

This experiment was designed to study the effects of pH on larval metamorphosis with or without tube using three pH treatments: pH 8.1, 7.9, and 7.7. Lower pH was not included in this experiment because the majority of larvae did not metamorphose with tube in the pH 7.6 treatment of Experiment 1. Embryos were cultured to the competent stage (in ~96 h) as described in Experiment 1. Each of the three pH treatments consisted of four replicate cultures and six replicate assay dishes per culture, each bioassay dish contained about 20–25 competent larvae. Larvae were placed in polystyrene Petri dishes (Falcon no. 1006) with 10 ml of FSW with corresponding pH levels. Bioassay dishes with larvae were kept in incubation chambers that were supplied with CO_2_-enriched air at the same levels as the treatment cultures for pH control. In each dish, there was a biofilmed (7-day-old assemblage of natural microbial community) coverslip to trigger larval metamorphosis and to serve as suitable hard substrate for larvae (Harder et al. 2002). Larvae were allowed to metamorphose on these biofilmed-coverslips for 24 h. During the bioassay, the pH generally decreased 0.2–0.3 units, irrespective of treatment conditions, likely due to larval calcification, respiration, and the microbial community (biofilm) used to induce larval metamorphosis. After 24 h, the number of individuals that were swimming, the number that were attached (metamorphosed without tube), and the number that had attached and had begun calcifying a tube were counted (metamorphosed with visible CaCO_3_ tube).

### Experiment 3: Effect of pH on post-settlement (juvenile) growth

This experiment was designed to examine the effects of pH ranging from 8.1 (control) to 7.5 on the growth rate of successfully metamorphosed and calcifying individuals. Since the majority of larvae in Experiment 2 failed to produce CaCO_3_ tubes at the time of metamorphosis at pH 7.7, here larvae were forced to metamorphose on a clean glass surface through mechanical stimulation (passing them through 300 micron mesh) and by keeping them at high density (e.g., 20 larval per ml) on a clean glass surface. Additionally, the pH during this longer (36 h) attachment and metamorphosis period was allowed to increase in the treatments (<0.3 pH unit increase). This method triggered the competent larvae to metamorphose and begin calcification, and >10 individuals were obtained per replicate culture tank to examine their post-settlement growth performance at different pH levels. These newly metamorphosed larvae were reared for 9 days in different pH seawater using *Isochrysis galbana* as their sole food source in culture conditions similar to the larvae. Juvenile growth rate was measured using the tube length as proxy after 1.5, 3, and 9 days. Lengths were measured by taking photos of >10 juveniles and measuring them using Image J photo analysis software. For each treatment, there were four replicate culture tanks, and for each tank, at least 10 individuals were measured.

### Data analysis

All data were tested for normality and homogeneity of variance with Shapiro–Wilk’s test and Cochran’s C-test, respectively. After meeting the assumptions of parametric analysis, embryonic and larval development data were analyzed using one-way ANOVA and repeated measure ANOVA (for all developmental stages). The slopes of the fitted linear regressions of juvenile growth rates were compared using an ANCOVA designed for comparing multiple slopes (Zar 1999). Since larval metamorphosis data did not meet parametric analysis assumptions even after data transformation, it was analyzed using nonparametric Kruskal–Wallis test.

## Results

### Carbonate chemistry variability due to pH treatment

Mean (±SD) values of the carbonate system in pH treatments are shown in Table 1. Treatment conditions were reliably stable within replicates (no effect of replicates, *F* < 0.5, *p* > 0.5 for all within treatment ANOVAs, Table 1), but treatment conditions did slightly change over time and among experiments possibly due to changes in CO_2_ pressure or changes in ambient levels of atmospheric CO_2_ during the experimental period. Nevertheless, the total alkalinity (TA) was approximately constant, ranging from 2,200 to 2,300 μmol l^−1^, irrespective of pH levels and experiments. Also, the treatment levels of *p*CO_2_ were close to the values used to calculate the target pH using theoretical values; control *p*CO_2_ = ~430–600 μatm, pH 7.9, *p*CO_2_ = ambient + 380–415 μatm, pH 7.7 *p*CO_2_ = ambient + 950–1,100 μatm, and pH 7.5 *p*CO_2_ = ambient + 1,947 μatm. These treatments are lower than yearly averages, with the lowest pH treatment going beyond seasonal fluctuations experienced by natural populations, which prefer the eastern oceanic waters of Hong Kong (mean pH 8.25, low pH 7.85) (Yung et al. 2001). CaCO_3_ mineral forms, calcite and aragonite, were saturated in all pH treatments with the exception of the pH 7.5 treatment in Experiment 3 that had the average aragonite saturation of Ωar = 0.76 (undersaturated, Table 1).

**Table 1:** Measured and calculated seawater carbonate system parameters for three experimental conditions used in this study

Parameter	Experiment 1		Experiment 2—development			Experiment 2—settlement assay			Experiment 3			
	Control	pH 7.6	Control	pH 7.9	pH7.7	Control	pH 7.9	pH 7.7	Control	pH 7.9	pH 7.7	pH 7.5
pH (NBS)	8.17 (±0.02)	7.56 (±0.02)	8.05 (±0.05)	7.85 (±0.13)	7.65 (±0.09)	7.95 (±0.07)	7.83 (±0.02)	7.73 (±0.04)	8.14 (±0.03)	7.91 (±0.06)	7.70 (±0.07)	7.49 (±0.04)
Total alkalinity (mM Kg^−1^)	2,232 (±92)	2,229 (±31)	2,290 (±150)	2,286 (±87)	2,307 (±82)	2,241 (±130)	2,320 (±38)	2,274 (±43)	2,201 (±15)	2,203 (±40)	2,170 (±27)	2,224 (±34)
pCO_2_ (μatm) (calculated)	425.3 (±25)	2,035.8 (±124)	604.7 (±111)	1,015.9 (±317)	1,691.0 (±419)	769.7 (±167)	1,085.1 (±80)	1,367.0 (±153)	455.3 (±39)	838.9 (±134)	1,404.6 (±240)	2,407.0 (±256)
Ωar (calculated)	2.99 (±0.12)	0.86 (±0.05)	2.44 (±0.43)	1.63 (±0.58)	1.08 (±0.31)	1.96 (±0.43)	1.59 (±0.10)	1.26 (±0.14)	2.78 (±0.18)	1.78 (±0.26)	1.13 (±0.20)	0.74 (±0.04)

The partial pressure of carbon dioxide (*p*CO_2_) and aragonite saturation level (Ωar) was calculated from total alkalinity and pH using CO2SYS (Pierrot et al. 2006)

### Embryonic and larval development (Experiment 1)

**Fig. 1:**
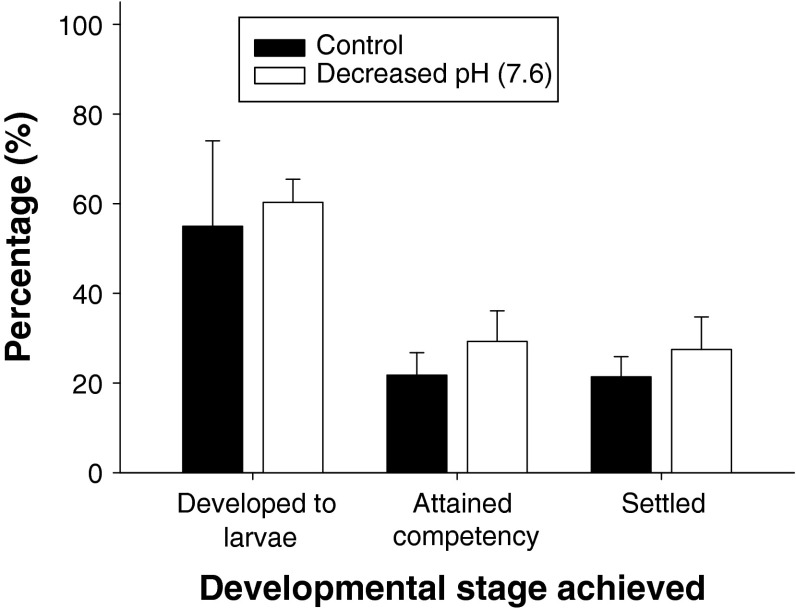
Effect of pH on the development of the tubeworm (*Hydroides elegans*): percentage of embryos reaching the larval stage, the competent stage, and settling after exposure to control or decreased pH treatment (pH 7.6)

About 50–60 % of embryos developed and hatched into trochophore larva within 16 h, regardless of pH treatment. There was no significant difference in embryonic development success between the treatment and the control (ANOVA, *F* = 0.83, *p* > 0.05). Although the mean larval survivorship was about 20 % at the end of the culture period, there was no significant difference between the treatment and the control (ANOVA, *F* = 2.93, *p* > 0.05), and the same was true when all the data were analyzed with a repeated measure ANOVA (*F* = 2.03, *p* > 0.05) (Fig. 1). Similarly, at the time of competence (to attach and metamorphose), larval total length (size) ranged between 247 and 278 μm and there was no significant difference between the treatment and the control (ANOVA, *F* = 1.91, *p* > 0.05).

### Larval metamorphosis (Experiment 2)

**Fig. 2:**
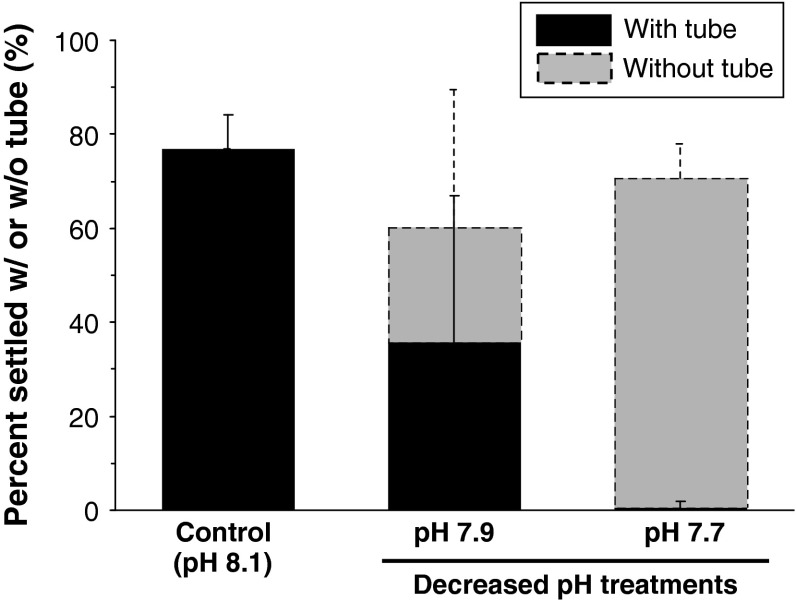
Effect of pH on larval settlement (or attachment) and metamorphosis in the tubeworm (*Hydroides elegans*): percentage of larvae reaching settlement with or without visible CaCO_3_ tube

The multi-species natural biofilm on glass induced ~80 % of larvae to attach and metamorphose with a CaCO_3_ tube within 24 h at the ambient pH level of 8.1 (control). Low pH treatments had no significant effect on percent larval metamorphosis (Kruskal–Wallis, *F* = 8.31, *p* > 0.05; Fig. 2). However, there was a significant effect of pH on the percentage (or proportion) of larvae that did or did not produce CaCO_3_ tube after metamorphosis (Kruskal–Wallis test results: H_withtube_ = 7.497; H_without tube_ = 5.692; *p* < 0.01; Fig. 2). Less than 30 % of larvae produced CaCO_3_ tube after metamorphosis at pH 7.9 (Fig. 2, middle bar), and almost none of the metamorphosed larvae produced CaCO_3_ tube in the pH 7.7 treatment.

### Early juvenile growth (Experiment 3)

**Fig. 3:**
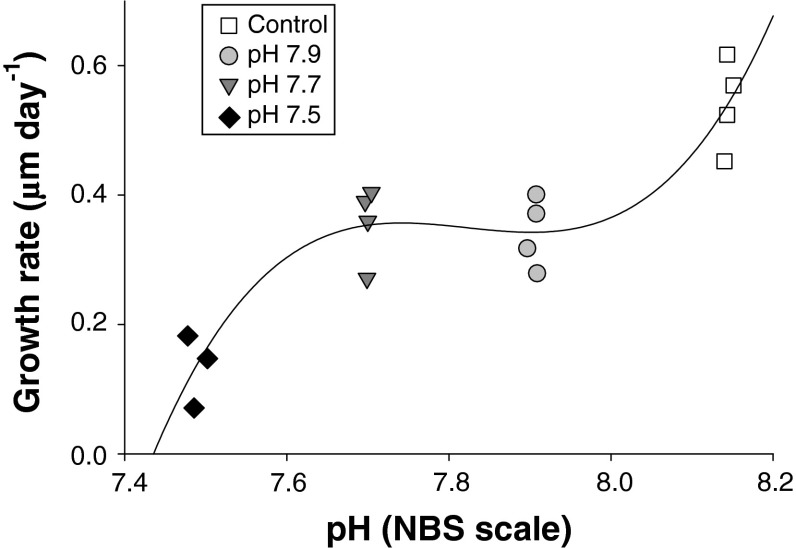
Effect of pH on post-settlement growth rate of the CaCO_3_ tube in the tubeworm (*Hydroides elegans*). Each symbol represents the average of 10 to 25 randomly selected individuals from one replicate. There were 3 to 4 replicates per pH treatment

Once metamorphosed, the post-settlement growth of CaCO_3_ tube length increased from ~0.5 mm on Day 1 (24 h after attachment) to ~4.5 mm on Day 9 in the ambient (control) pH 8.1 (Fig. 3). Low pH had strong and significant negative effects on post-settlement growth rate (ANCOVA: *F* = 59.01, *p* < 0.001, Fig. 3). The multiple comparison tests showed three distinct juvenile groups on Day 9: the control pH 8.1 (~4.5 mm), pH 7.9 and 7.7 (~3 mm), and pH 7.5 (~1 mm) (Fig. 3). The best fit trend line (*R* ^2^ = 0.874) illustrates the differences (and similarities) between groups, only pH 7.5 experienced aragonite undersaturation (Fig. 3).

## Discussion

In recent years, the impact of ocean acidification on larval growth and calcification has been the focus of many research projects (e.g., Kurihara 2008; Dupont and Thorndyke 2009; Dupont et al. 2010; O’Donnell et al. 2010; Talmage and Gobler 2010; Byrne 2011; Gazeau et al. 2011). However, the impact of this emerging stressor on the larval metamorphosis of economically important biofouling species has been elusive (McDonald et al. 2009) and thereby precludes our ability to predict or understand biofouling intensity in future ocean conditions (Poloczanska and Butler 2010). This study clearly demonstrates that metamorphosing larvae of the tubeworm, *Hydroides elegans*, suffer greatly at pH levels projected to occur in the coming century (pH 7.7). Even if metamorphosis is successful (with tube), subsequent calcification and tube growth were significantly decreased in the low pH treatments projected to occur within this century (pH 7.9). Gregarious tube building polychaete worms create a unique habitat that houses many other marine invertebrates, and so the decrease in tube length will lead to decreased availability of heterogeneous hard substrates created by them, potentially leading to a restructuring of communities that depend on these ecosystem engineers (Poloczanska and Butler 2010). Thus, within this century, this ecologically (i.e., pioneering ecosystem engineer) and economically (i.e., biofouling) important species will be greatly threatened due to OA. Long-term consequences of impaired larval metamorphosis and early growth on population structure and dynamics are not yet known, but the reduced ability of postlarvae to calcify and decreased juvenile growth indicates that this species will be under severe stress in the coming century.

### Larval development in low pH seawater (or in high-CO_2_ coastal oceans)

The “soft” trochophore larval stage of the tubeworm does not have structures that are dependent on calcification such as a CaCO_3_ shell or carapace and may not need to increase efforts to calcify as carbonate ion concentrations decrease. Therefore, they may be expected to suffer less detriment due to low pH environments than their calcifying counterparts in the phylums Mollusca and Echinodermata, that is, >75 % larvae did not develop at pH lower than 7.6 (Kurihara et al. 2007; Dupont et al. 2008; Clark et al. 2009; Miller et al. 2009; O’Donnell et al. 2009; Parker et al. 2009; Talmage and Gobler 2009; Watson et al. 2009; Byrne 2011). This hypothesis is supported by this study because larval development, growth, and survival were not significantly affected by low pH. Fertilized eggs exposed to control and low pH conditions developed and settled with no significant differences (Fig. 1).The sensitivity of “soft” larval stages (including their embryos) to low pH is highly species-specific (Byrne 2011; Byrne et al. 2011), but most of the examined species, like this tubeworm, showed high relative tolerance to low pH during their pelagic developmental period. For example, similar to the non-effect seen in our experiments and contrary to calcifying larvae, >75 % of embryo and larvae of several cnidarian species successfully developed in pH as low as 7.3 (Kurihara 2008; Suwa et al. 2010). However, the non-calcifying nature of the *H. elegans* larvae may not explain the apparent tolerance to low pH. Lecithotrophic life histories, like those of the cnidarians mentioned above, may be more robust to environmental stressors than their planktotrophic counterparts (Dupont et al. 2010); while this may be due to decreased environmental interaction, it is possible that the tolerance observed in this polychaete may be explained by some other, unmeasured parameter (e.g., increased metabolism or feeding rates). Additionally, the larvae of another tube worm *Pomatoceros lamarckii* suffered greatly at decreased pH (pH 7.6 and 7.4) (Lewis et al. 2012). The resilience of the pelagic larval stages of *H. elegans* to low pH may also be related to a preadapted ability to survive in a variety of unstable and unfavorable environments (Whiteley 2011). This tubeworm is found worldwide in oceanic and estuarine habitats, experiencing a wide range of environments with fluctuating pH’s, salinities, and temperatures (Qiu and Qian 1997), indicating that they may have been preselected to cope with stressors like low salinity, high temperature, and possibly low pH.

Although larval morphological features appeared to be unaffected by low pH over their developmental stages, physiological processes are expected to be, and are, affected by low pH in several invertebrates (Widdicombe and Spicer 2008; Dupont and Thorndyke 2009). Such altered physiological fitness traits may contribute to decreased fitness or reduced energy reserves potentially observable in performance parameters other than survival and growth (Gaylord et al. 2011) such as altered metabolism (Michaelidis et al. 2005; Nakamura et al. 2011; Stumpp et al. 2011b) and reduced energy reserves (Talmage and Gobler 2010). Therefore, it is recommended that future studies should examine these physiological traits along with larval development to gain further new insights into pH effects on larval energetic balance (Clark et al. 2009; Nakamura et al. 2011).

### Larval metamorphosis in low pH seawater

One of the main aims of this study was to determine how the tubeworm larval metamorphosis is exercised in a range of pH conditions. The majority of competent larvae, >75 %, successfully attached and metamorphosed at pH levels as low as 7.6 (Figs. 1, 2). Larval attachment and subsequent metamorphosis, up to branchial lobe formation, in *H. elegans* do not require extensive gene translation or production of new tissues or organs (Carpizo-Ituarte and Hadfield 1998; Nedved and Hadfield 2009). This may contribute to why the attachment and initial metamorphosis can be predicted to be less sensitive to low pH. Surprisingly, however, at an environmentally and climatically realistic pH level of 7.7, almost none of those metamorphosed individuals produced a CaCO_3_ tube during the 24-h settlement assay. The reasons for this inability to build a visible CaCO_3_ tube at the time of metamorphosis in low pH conditions cannot be determined from our study, but may be related to (1) altered physiological mechanisms such as metabolism may result in decreased ability to calcify at decreased pH (Metzger et al. 2007; Ellis et al. 2009), (2) the exposure to decreased pH stress during their pelagic development might also have decreased their physiological fitness at the time of metamorphosis, due to the latent, or carryover, effects (Marshall et al. 2003; Dupont et al. 2012), and (3) unfavorable carbonate chemistry conditions to build tube at the site of metamorphosis, for example, lowered carbonate ion concentrations and/or CaCO_3_ mineral saturation states make calcification difficult. Latent effects can manifest in a variety of ways potentially resulting in impaired calcification-related signaling pathways, due to changes in gene and/or protein expression (Tomanek et al. 2011). Recent studies suggest that the response of calcification-specific genes to pH is highly species-specific, that is, down-regulated in larval sea urchin of the *Lytechinus pictus* (O’Donnell et al. 2010), the *Strongylocentrotus purpuratus* (Stumpp et al. 2011a), and up-regulated in the larval sea urchin *Paracentrotus lividus* (Martin et al. 2011). Nevertheless, molecular mechanisms underlying decreased pH impacts on calcification and/or other associated physiological processes are still a black box (Hofmann et al. 2008; Tomanek et al. 2011).

The discussion on possible sources of the inability to calcify can be expanded upon further when the third experiment to measure juvenile growth in decreased pH conditions is considered. In the Experiment 3, the stimulation by use of violent rinsing, overcrowding, brief pH relief or the 36-h period (as opposed to 24 h) allowed for settlement enabled the larvae to calcify, despite the decreased pH of the treatments in which the larvae were reared. Future experiments will need proper controls to determine the exact inducer of the calcification, but assuming mechanical stimulation, overcrowding, and/or pH relief did induce the construction a CaCO_3_ tube, we are lead to the possibility that the inability to calcify normally is not caused by the calcification process being physically incapable of calcifying in decreased concentrations of carbonate ions. Secondly, the latent effects of development in decreased pH seawater may be reversible to some degree. The inducers may have inspired an increased metabolic effort, or potentially brought about a stress response that is not expressed when pH is the only stressor. The ability to quickly induce calcification would indicate that pH stress may be combated with a preexisting response that is not elicited by pH stress alone. Our experiment, however, does not account for the possibility that the different batches of larvae respond differently to pH stress.

### Post-settlement growth in low pH seawater

Low pH conditions also dramatically retarded the post-settlement growth of CaCO_3_ tubes (Fig. 3); for instance, the mean tube growth in the low pH 7.5 treatment was less than one-third that of the control. The tubeworm *H. elegans*, like several other serpulid polychaetes, calcifies a bimineralic tube that contains comparable proportions of the two most common polymorphs of calcium carbonate, aragonite and calcite (Chan et al. 2012). The proportion of calcite to aragonite is highly variable in tubeworms, for example, *H. norvegicus*, 98_Cal_:2_Ar_; *H. dianthus*, 60_Cal_:40_Ar_ (Tanur et al. 2010; Taylor et al. 2010). However, tubes of the newly metamorphosed *H. elegans* are dominated by the more soluble aragonite (Chan et al. 2012). Undersaturated levels of aragonite, in the pH 7.5 treatment, during early juvenile growth may therefore suppress the calcification by promoting dissolution of the aragonitic tube or making the initial formation of aragonite more difficult. Additionally, because the acquisition of the individuals in this experiment may have selected for relatively strong genotypes, for example, those able and ready to metamorphose immediately and under stressful conditions, the measurements here may underestimate the effects of low pH on growth. Nevertheless, the poor calcification and subsequent depressed growth of calcified structures observed here are common symptoms of low pH conditions as previously observed (Findlay et al. 2009; Talmage and Gobler 2010). Likewise, a similar study encompassing the larval and juvenile period of a barnacle (McDonald et al. 2009) observed decreased juvenile shell strength, a similar finding as the larval stages were unaffected while juvenile calcification was altered in both *H. elegans* and *Balanus amphitrite*.

Tube length in *H. elegans* is related to its fitness, as it reflects individual size, and hence, reproductive output; smaller individuals produce fewer and low-quality gametes (Qiu and Qian 1997). Not only will these smaller individuals produce fewer offspring because of their size, but the pH stress that lead to the smaller size, if persistent, can be expected to place continued pressure on these animals and reduce reproductive output further than would be expected solely based on their smaller size. Decreases in reproductive output could result in reduced larval supply, potentially shrinking population sizes. Furthermore, aggregations of *H. elegans* can form large masses of highly diverse solid substrate, transforming what would otherwise be a relatively uninhabitable two-dimensional surface into a topographically diverse habitat that provides shelter for many animals and hugely increases the biofouling intensity (or biomass). Smaller animals with shorter tubes may lead to reductions in aggregation biomass and stability which in turn will affect the communities that utilize these aggregations, which prefer the three-dimensional structures of the aggregations to the otherwise smooth surfaces which *H. elegans* commonly colonize.

### Calcification strategies and their role in low pH tolerance

Larvae of sessile calcifying invertebrates are exposed to the pelagic environment and therefore dependent on the environmental conditions for survival, leading to the “vulnerable larvae” theory (Pechenik 1999). The adult forms, on the other hand, have protective shells into which they may withdraw during stressful periods, potentially managing stress levels through physiological and/or behavioral changes. However, when considering a persistent stress like low pH, exposure is permanent and differences in an organisms’ physiological requirements may preempt the benefits of energetically expensive shells. For example, while non-calcifying coral larvae were not affected by low pH, adult calcification decreased (Albright et al. 2008; Suwa et al. 2010). While decalcification in adult corals may not be lethal (Fine and Tchernov 2007), it is clear that the other life stages may respond differently to decreased pH. Calcifiers that are highly dependent on their calcified structures may require a large energy investment, so it has been hypothesized that these obligatory calcifiers will invest more energy in calcification to compensate, thereby reducing energy available for growth and reproduction (Melzner et al. 2011; Stumpp et al. 2011b). Like corals, other calcifiers have different life history stages with different calcification strategies (e.g., barnacles, tubeworms, and bryozoans), but few studies compare these life stages. For example, the only effect of low pH (pH 7.4) on the barnacle *Balanus amphitrite* was observed in shell strength of juveniles, larvae developed, and metamorphosed normally (McDonald et al. 2009). Likewise, the observations of the tubeworm *H. elegans*, presented here, showed that larvae appeared tolerant while juvenile calcification was reduced. Contrarily, copepods, which use a similar calcification method to barnacles but do not produce large calcitic shells, performed well throughout their entire life cycle (Mayor et al. 2007; Kurihara and Ishimatsu 2008). However, a few studies provide evidence that calcification strategy is not the only determinant regarding pH tolerance, for example, lecithotrophic larvae and calcifying juveniles of the sea star *Crossaster papposus* increased growth in response to pH (Dupont et al. 2010), one coral’s non-calcifying larvae (*Acropora digitifera*) experienced reduced metamorphosis at low pH (Nakamura et al. 2011), and more directly relevant to this research, Lewis et al. (2012) observed decreased larval survival in response to low pH despite the non-calcifying nature (*Pomatoceros lamarckii*). The potential importance of calcification strategy during larval stages complicates the “vulnerable larvae” hypothesis, while more vulnerable to many other stressors and predation, certain larval forms may be better suited for the future low pH conditions.

### Long-term shifts toward stressful low pH environments

Predicting the long-term impact of decreased pH is not as simple as exposing extant animals to the pressures of future oceans, it must be considered that animals may adapt over coming centuries. While *H. elegans* clearly suffers detrimental effects at decreased pH levels expected to occur in the next 50–100 years, this species has the potential to go through multiple generations each year. A very conservative estimate would place at least 50 generations between now and the “future” high CO_2_ future tested in this study, a number well beyond what is necessary for natural selection (Carroll et al. 2007). The *H. elegans* is capable of living in coastal where pH, salinity, and temperature are notoriously variable, and these periodically stressful conditions may play a role in maintaining genetic variability, variability that may now be selected from as environmental stressors like pH begin to shift in one direction (Hoffmann and Hercus 2000). Sunday et al. (2011) examined maternal and paternal influence on development in an urchin and a mussel, Pistevos et al. (2011) compared differences between colonies of a bryozoan, and Foo et al. (2012) observed significant environment by genetic interactions, all three studies found variable responses to pH (or temperature) between genetically related groups (clones or siblings) indicating some degree of heritable variation in pH response that may be selected for over the coming centuries. Adaptation over the coming century, especially in animals such as *H. elegans* with short generation times, should be expected and needs to be examined whether accurate forecasts of future ecosystems are to be made.

## Conclusions

This study examined the effects of pH on larval development, attachment, and metamorphosis in a species whose phyla is greatly underrepresented in climate change and ocean acidification research to date. It is shown that the common biofouling tubeworm, *H. elegans*, faces a great challenge when subjected to decreased pH conditions with symptoms like metamorphosing without a protective tube and decreased juvenile growth. As a major component of shallow subtidal fouling communities, reductions in their growth and biomass could lead to shifts in the composition, intensity, and function of the fouling community. Thus, our findings support the argument that “fouling communities, composed largely of calcifying organisms and organism with highly sensitive larval stages, will suffer decreases in biomass under acidified conditions or force shifts toward communities composed of non-calcifying, OA tolerant, species” (Poloczanska and Butler 2010). Other major calcifying biofoulers are also highly sensitive to decreased pH, for example, mussels have shown decreased growth and reduced immune function (Michaelidis et al. 2005; Bibby et al. 2008) and barnacles are more likely to suffer as well as a consequence of delayed and smaller broods (Findlay et al. 2010); however, the changes elicited when only low pH is examined may be overcome when other conditions are met, such as when the mussel *Mytilus edulis* had sufficient food, the effects of low pH were reduced (Melzner et al. 2011). According to this study, fouling communities, especially those that are composed of the highly soluble form of CaCO_3_ (aragonite), for example, the tubeworm *H. elegans*, may be affected by the shifts in seawater carbonate chemistry system expected for the coming centuries due low pH. However, without studies combining the effects of food supply and other stressors, it is impossible to truly predict how this species will respond. It is therefore important to biofouling research, in addition to ecologically focused OA research, to understand how biofouling community will be affected by decreased pH and what other factors may play a role in exacerbating or relieving those harmful effects.
